# A suspected case of drug-induced tubulointerstitial nephritis by pilocarpine hydrochloride

**DOI:** 10.1007/s13730-019-00401-8

**Published:** 2019-05-10

**Authors:** Teruhiro Fujii, Kentaro Kawasoe, Yuki Nishizawa, Jumpei Kashima, Akiko Tonooka, Akihito Ohta, Kosaku Nitta

**Affiliations:** 1 Division of Nephrology, Department of Medicine, Tokyo Metropolitan Komagome Hospital, 3-18-22, Honkomagome, Bunkyo-Ku, Tokyo, 113-0021 Japan; 2 Division of Pathology, Department of Medicine, Tokyo Metropolitan Komagome Hospital, 3-18-22, Honkomagome, Bunkyo-Ku, Tokyo, 113-0021 Japan; 3 Department IV, Internal Medicine, Tokyo Women’s Medical University, 8-1, Kawadacho, Shinjuku-Ku, Tokyo, 162-8666 Japan

**Keywords:** Pharyngeal cancer, Pilocarpine, Tubulointerstitial nephritis

## Abstract

A 63-year-old man with pharyngeal cancer had been prescribed pilocarpine hydrochloride for xerostomia after concomitant chemoradiotherapy. After 6 months of taking pilocarpine hydrochloride, he was referred to our hospital due to gradually developing renal insufficiency. The patient underwent detailed urinalysis, blood chemistry analysis, immune-serology testing. A renal biopsy was also performed. He was diagnosed with chronic tubulointerstitial nephritis (TIN) caused by lymphocytic infiltration of the interstitium, tubular atrophy, and interstitial fibrotic changes. Infections, autoimmune diseases, and genetic factors were ruled out as causes of TIN; a drug-induced lymphocyte stimulation test confirmed that he had high stimulation index scores for pilocarpine hydrochloride and a normal range stimulation score for other supplements. These results indicated that the TIN could have been induced by pilocarpine hydrochloride. Drug discontinuation partly improved his renal function and tubule marker levels. To our knowledge, this is the first report of TIN following administration of pilocarpine hydrochloride. This finding could contribute to future treatment decisions for patients with TIN and those using pilocarpine hydrochloride.

## Introduction

Pilocarpine hydrochloride, a cholinergic agonist, is used for treating xerostomia associated with radiotherapy for head and neck cancer and for Sjögren’s syndrome [1–3]. Common side effects include miosis, hyperhidrosis, nausea, liver dysfunction, diarrhea, frequent urination; serious side effects include interstitial pneumonia and loss of consciousness [4]. However, nephrotoxicity by pilocarpine hydrochloride has to date, not been reported. Tubulointerstitial nephritis (TIN) is a renal histologic lesion characterized by the presence of inflammatory infiltrates and edema within the tubulointerstitial compartment, which usually does not affect the glomerular and vascular compartments [5]. Any drug can cause TIN; hence, clinical and laboratory findings, including renal biopsy, are important for diagnosis [6]. We report, herein, a rare instance of pilocarpine hydrochloride-induced TIN.

## Case report

A 63-year-old man was admitted to the Tokyo Metropolitan Komagome Hospital because of renal insufficiency. The patient had received insulin treatment since the age of 40 years for type 2 diabetes mellitus. He was treated with external beam radiation 70 Gy radiation therapy along with concomitant chemotherapy consisting of 100 mg/m^2^ cisplatin once every 3 weeks three times in total for pharyngeal carcinoma since the age of 62 years. He had no history of allergies or any other significant medical history.

Following concomitant chemotherapy, he had been prescribed pilocarpine hydrochloride for xerostomia associated with radiotherapy for head and neck cancer. In addition, he was taking four types of supplements (zinc, vitamin E, branched-chain amino acids, and docosahexaenoic acid) every day since the age of 61. When pilocarpine hydrochloride therapy commenced, the patient’s creatinine (Cr) level was 1.0 mg/dL. Subsequently, renal dysfunction gradually developed, and his Cr rose to 2.05 mg/dL after 6 months of treatment. The patient was hospitalized for intensive examination and to treat his renal insufficiency.

Upon admission, the patient weight and height were 63 kg and 177 cm, respectively. The patient’s blood pressure was 139/86 mmHg, pulse rate was 58 beats/min, and body temperature was 36.3 °C. Chest, heart, and abdominal findings were unremarkable. Ophthalmological examination indicated no uveitis, and no superficial lymphadenopathies or rashes were observed. The electrocardiogram showed no significant abnormal findings. Ultrasonography revealed an enlarged kidney. There were no findings suggestive of a post-renal lesion on computed tomography. However, gallium scintigraphy and positron emission tomography were not performed due to their low specificity.

Laboratory findings on admission are shown in Table 1. Urinary findings indicated the absence of proteinuria or hematuria but the presence of leukocyturia [10–19 white blood cells/high-power field (HPF)], and urinary excretion of β2-microglobulin (β2MG) was 1542 μg/gCr. The white blood cell (WBC) count and hemoglobin (Hb) level were 5100 cells/μL and 11.5 g/dL, respectively. Blood eosinophils accounted for 3.9% of circulating lymphocytes. The results of blood biochemistry tests indicated that blood urea nitrogen (BUN) was 30.4 mg/dL, and Cr was 2.05 mg/dL. Electrolyte and liver function tests were normal. Blood sugar levels and hemoglobin A1c (HbA1c) were under control. Immunological tests for C-reactive protein (CRP), myeloperoxidase antineutrophil cytoplasmic antibody (MPO-ANCA), proteinase 3 antineutrophil cytoplasmic antibody (PR3-ANCA), antinuclear antibody (ANA), anti-SS-A/Ro antibodies, and anti-SS-B/La antibodies were all negative.

**Table 1:** Laboratory findings on admission

Urinalysis			Blood chemistry			Immuno-serology		
Urinometry	1.011		TP	6.6	g/dL	CRP	0.06	mg/dL
pH	5.0		Alb	4.1	g/dL	IgG	862	mg/dL
Protein	–		BUN	30.4	mg/dL	IgA	126	mg/dL
Occult blood	–		Cr	2.05	mg/dL	IgM	63	mg/dL
β2MG	1542	μg/gCr	UA	8.4	mEq/L	C3	85	mg/dL
NAG	26.1	IU/gCr	Na	140	mEq/L	C4	33.5	mg/dL
			K	4.3	mEq/L	CH50	55.8	U/mL
Urine sediment			Cl	109	mEq/L	ANA	< 40 ×	
RBC	<1/HPF		Ca	9.1	mg/dL	Anti-SS-A antibody	Negative	
WBC	10-19/HPF		iP	3.4	mg/dL	Anti-SS-B antibody	Negative	
Cast	–		AST	26	IU/L	MPO-ANCA	< 1.0	U/mL
			ALT	23	IU/L	PR3-ANCA	< 1.0	U/mL
Hematology			LDH	193	IU/L			
WBC	5100	/μL	ALP	206	IU/L	DLST	SI	Normal range (< 180%)
Neutrophil	76.6	%	Glu	88	mg/dL	Supplement 1 (zinc)	49%	
Eosinophil	3.9	%	HbA1c	6.2	%	Supplement 2 (vitamin E)	135%	
Monocytes	11.7	%				Supplement 3 (branched-chain amino acids)	78%	
Lymphocytes Hb	7.6 11.5	% g/dL				Supplement 4 (docosahexaenoic acid)	158%	
Plt	21.5 × 10^4^	/μL				Pilocarpine hydrochloride	290%	

β2MG, β2-microglobulin; NAG, *N*-acetyl-β-d-glucosaminidase; RBC, red blood cell; WBC, white blood cell; HPF, high power field; Hb, hemoglobin; Plt, platelet; TP, total protein; Alb, albumin; BUN, blood urea nitrogen; Cr, creatinine; UA, uric acid; Na, sodium; K, potassium; Cl, chloride; Ca, calcium; iP, inorganic-phosphate; AST, aspartate transaminase; ALT, alanine transaminase; LDH, lactate dehydrogenase; ALP, alkaline phosphatase; Glu, glucose; HbA1c, hemoglobin A1c; IgG, immunoglobulin G; IgA, immunoglobulin A; IgM, immunoglobulin M; C3, complement 3; C4, complement 4; CH50, complement hemolytic activity; ANA, anti-nuclear autoantibody; anti-SS-A antibody, anti-Sjögren’s syndrome-A antibody; anti-SS-B antibody, anti-Sjögren’s syndrome-B antibody; MPO-ANCA, myeloperoxidase antineutrophil cytoplasmic antibody; PR3-ANCA, proteinase 3 antineutrophil cytoplasmic antibody; DLST, drug lymphocyte stimulation test; SI, stimulation index

**Fig. 1:**
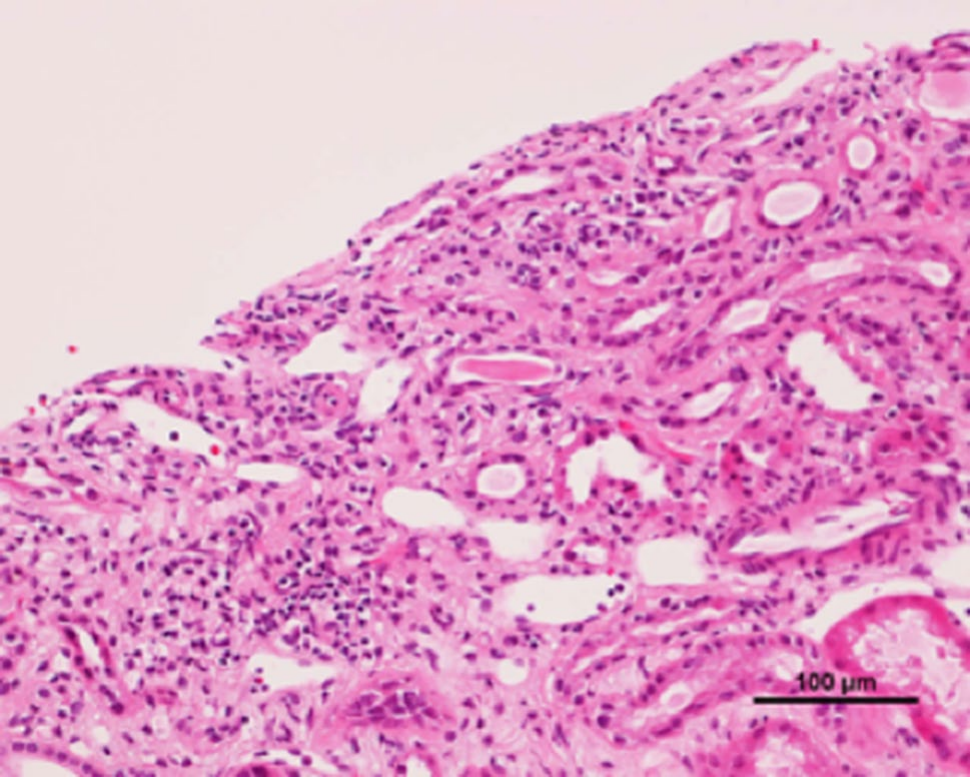
Light microscopic findings of renal biopsy. Renal biopsy demonstrates interstitial inflammatory infiltration and thinning of renal tubular epithelium (hematoxylin/eosin stain)

**Fig. 2:**
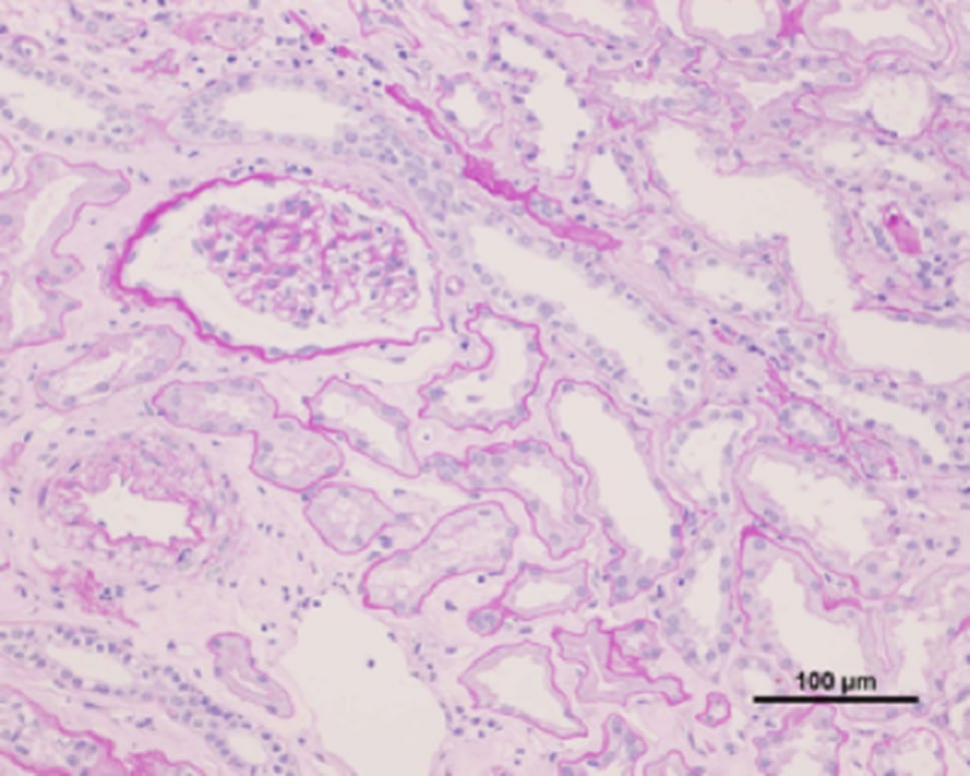
Tubular atrophy and interstitial fibrotic changes were notable, along with dilatation of some tubules, mild infiltration of mononuclear cells, and very few tubular casts (periodic acid–Schiff stain)

**Fig. 3:**
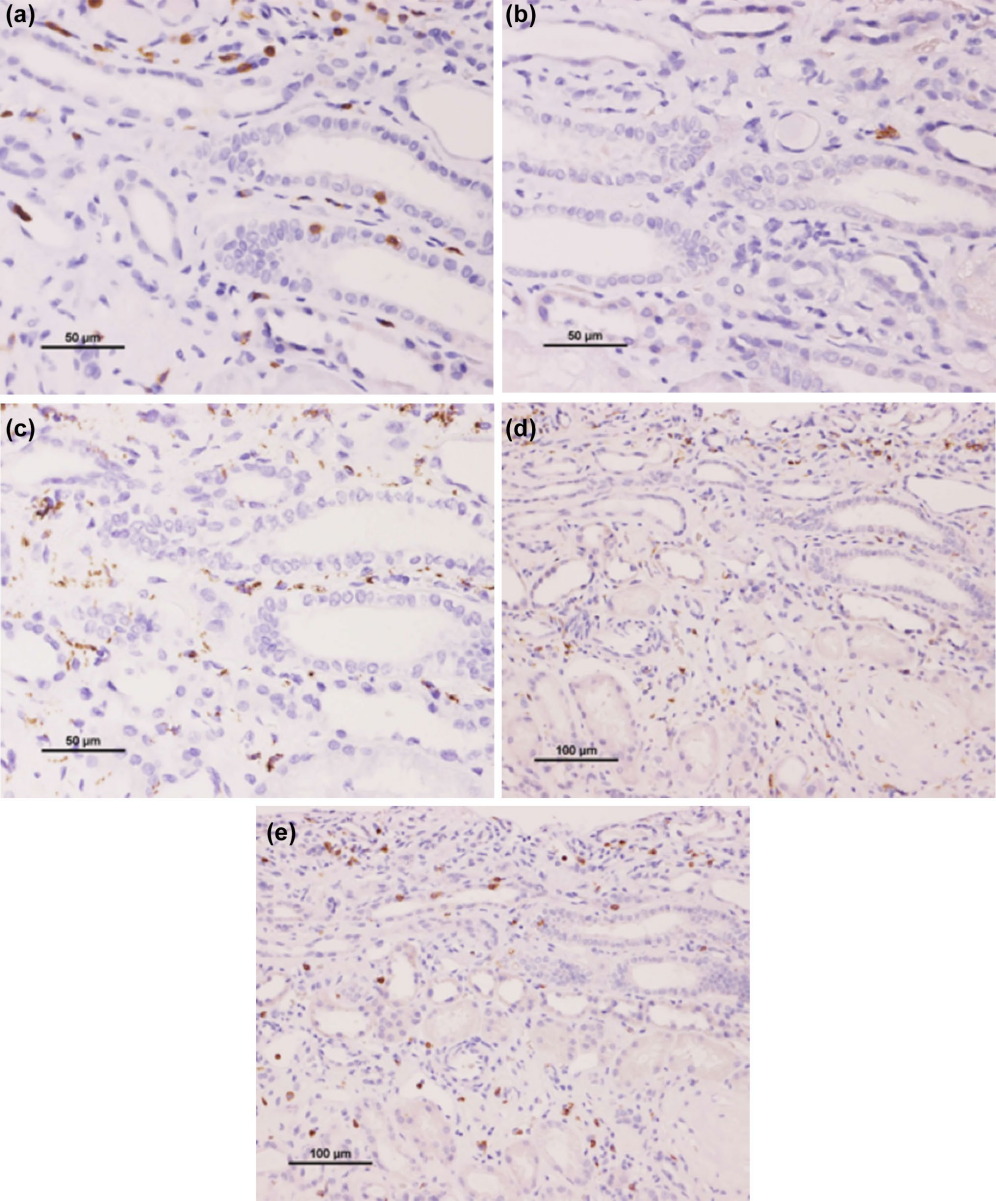
Immunohistochemistry showed CD3 expression in interstitium and arrow shows the infiltration in the tubular epithelia. CD20 and CD68 staining did not show infiltration compared to CD3 staining (**a**–**c**). CD4 and CD8 were equally expressed (**d**, **e**)

**Fig. 4:**
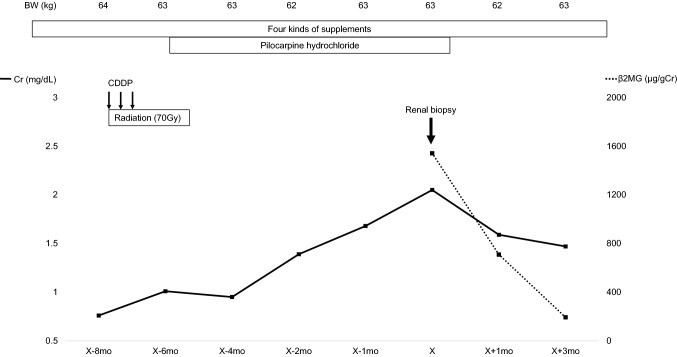
Clinical course of the patient over a period of 12 months. X represents the point of renal biopsy. BW, body weight; Cr, creatinine; CDDP, cisplatin; β2MG, β2-microglobulin; mo, month

A renal biopsy was performed to assess renal insufficiency. The sample of renal cortex contained fourteen glomeruli, three of which showed global sclerosis, and the remaining eleven glomeruli were borderline normal. Moderate tubular injury, mild interstitial inflammation, and thinning of renal tubular epithelium were observed (Fig. 1). Tubular atrophy and interstitial fibrotic changes were notable, along with dilatation of some tubules, mild infiltration of lymphocyte, and very few tubular casts (Fig. 2). Immunofluorescent staining for IgG, IgA, IgM, C3, and C1q yielded negative results. Immunohistochemical analysis showed CD3 expression in the interstitium and tubular epithelial infiltration, indicating tubulitis (Fig. 3a). In contrast, CD20 and CD68 staining was not as prominent as CD3 staining, demonstrating lower expression (Fig. 3b, c). CD68 was expressed mildly in the interstitium. CD4 and CD8 were equally expressed (Fig. 3d, e). These findings were consistent with those of chronic TIN. A drug-induced lymphocyte stimulation test (DLST) confirmed that the patient had a negative stimulation index (SI) score for the four supplements, whereas he had a high SI score of 290% for pilocarpine hydrochloride (the cut off value for DLST positivity is 180%). Drug-induced TIN, especially pilocarpine hydrochloride-induced TIN was suspected, due to the lack of suggestive findings of ocular disease by ophthalmological screening in addition to the laboratory and pathological results, and the patient’s clinical history. The patient, therefore, discontinued pilocarpine hydrochloride. After drug discontinuation, his Cr level partly recovered from 2.05 to 1.47 mg/dL and his tubule marker level also improved (Fig. 4), and the kidney returned to normal size.

## Discussion

TIN is a renal histologic lesion characterized by the presence of inflammatory infiltrates and edema within the tubulointerstitial compartment but usually does not affect the glomerular and vascular compartments [5]. TIN can be caused by medications; infections including bacteria, fungi, and viruses; systemic diseases (Sjögren’s syndrome, TIN with uveitis syndrome, immunoglobulin G4-related diseases); and idiopathic forms of diseases. Drug-induced TIN accounts for most cases and antibiotics represent the most common class of drug used for responding to TIN followed by proton-pump inhibitors and non-steroidal anti-inflammatory drugs (NSAIDs). However, a wide variety of other medications have been implicated. The clinical presentation is highly varied with the classic triad of fever, rash and eosinophilia occurring in a small minority of patients. Thus, renal biopsy is required for a definitive diagnosis [7].

The clinical diagnosis of drug-induced TIN is quite challenging since numerous types of medications have been implicated in causing TIN. However, the diagnosis of TIN may be aided by laboratory examinations and these can include DLST, which measures the proliferation of T cells induced by an agent in vitro, leading to suspicion of previous sensitization in vivo. DLST has been regarded as a useful diagnostic procedure for drug hypersensitivity, including drug-induced TIN [8]. Although, there are diseases and drugs that tend to cause low sensitivity and/or false positives [9], DLST may be effective when diagnosing drug-induced interstitial nephritis with less well-known drugs and patients with autoimmune diseases that cause interstitial nephritis [10].

Our patient was ruled out as having an infection or any systemic diseases; dehydration due to body weight variations (Fig. 4) was also ruled out. However, the patient showed high scores for pilocarpine hydrochloride in the DLST and as such, we ascribed his renal dysfunction to drug-induced TIN. We also considered the possibility of CDDP nephrotoxicity as chronic use of CDDP can cause tubular atrophy and interstitial fibrosis [11]. However, CDDP is not known to cause interstitial nephritis [12] and, in our case, the usage period was short and probably not sufficient to induce those histological changes. There is no standard approach for treating drug-induced TIN. The consensus is to withdraw the responsible medication as soon as possible [13]; any delay in drug withdrawal can adversely affect kidney function recovery. The role of steroids in treating TIN is not clear and there are no randomized controlled studies to guide therapy [14]. Several studies have reported that steroids appear to have some beneficial role, but Muriithi et al. found increased amounts of interstitial fibrosis and tubular atrophy with smaller kidneys evident on ultrasound, correlated with a poor response to steroids [15]. Steroids should be avoided in patients with advanced fibrosis, particularly in those with diabetes and labile blood glucose. In our case, we did not opt for steroid therapy due to interstitial fibrosis evident in renal biopsy and the risk of detrimentally affecting of glycemic control.

Kidney function in our case was only partly recovered. Two reasons can be considered for the only partial improvements in renal function. First, drug-induced TIN was diagnosed 6 months after the introduction of pilocarpine hydrochloride. The long delay between pilocarpine hydrochloride introduction and TIN diagnosis could explain the partial recovery of kidney functionality. Second, our patient had mild renal dysfunction before developing renal dysfunction. When he was initially treated with cisplatin for pharyngeal cancer, his renal function deteriorated temporarily. After chemotherapy, his Cr slightly worsened to 1.0 mg/dL (baseline 0.8 mg/dL) by cisplatin nephrotoxicity.

This is the first reported case of TIN induced by pilocarpine hydrochloride. A thorough assessment including the assessment of medication history, DLST, and renal biopsy was helpful in establishing the diagnosis in our patient who had a complex past renal history.
